# Shea Butter Potentiates the Anti-Bacterial Activity of Fusidic Acid Incorporated into Solid Lipid Nanoparticle

**DOI:** 10.3390/polym14122436

**Published:** 2022-06-16

**Authors:** Heba S. Elsewedy, Tamer M. Shehata, Wafaa E. Soliman

**Affiliations:** 1 Department of Pharmaceutical Sciences, College of Clinical Pharmacy, King Faisal University, Alhofuf 36362, Al-Ahsa, Saudi Arabia; tshehata@kfu.edu.sa; 2 Department of Pharmaceutics, College of Pharmacy, Zagazig University, Ash Sharqiyah, Zagazig 44519, Egypt; 3 Department of Biomedical Sciences, College of Clinical Pharmacy, King Faisal University, Alhofuf 36362, Al-Ahsa, Saudi Arabia; weahmed@kfu.edu.sa; 4 Department of Microbiology and Immunology, Faculty of Pharmacy, Delta University for Science and Technology, Gamasa, Mansoura 11152, Egypt

**Keywords:** topical delivery, optimization, shea butter, fusidic acid, solid lipid nanoparticle, anti-bacterial activity

## Abstract

Fusidic acid (FA) is an efficient anti-bacterial drug proven to be efficient against a wide range of bacteria. Nevertheless, the main restriction in its formulation is the limited solubility. To avoid such an obstacle, the drug is incorporated into the lipid core of the nanolipid formulation. Consequently, the present study was an attempt to formulate nanolipid preparation, mainly, solid lipid nanoparticle (SLN) integrating FA. FA-SLN was prepared using shea butter as a lipid phase owing to its reported anti-bacterial activity. Different FA-SLNs were fabricated using the central composite design (CCD) approach. The optimized formula was selected and integrated into a hydrogel base to be efficiently used topically. FA-SLN-hydrogel was evaluated for its character, morphology, in vitro release and stability. The formula was examined for irritation reaction and finally evaluated for its anti-bacterial performance. The optimized formula showed particle size 283.83 nm and entrapment 73.057%. The formulated FA-SLN-hydrogel displayed pH 6.2, viscosity 15,610 cP, spreadability 51.1 mm and in vitro release 64.6% following 180 min. FA-SLN-hydrogel showed good stability for three months at different conditions (room temperature and refrigerator). It exhibited no irritation reaction on the treated rats. Eventually, shea butter displayed a noteworthy effect against bacterial growth that improved the effect of FA. This would indicate prospective anti-bacterial activity of FA when combined with shea butter in SLN formulation as a promising nanocarrier.

## 1. Introduction

Skin as the largest organ in the human body is considered one of the mechanical barriers that provide a defense mechanism against pathogens and foreign bodies [1]. The most common route of delivering drugs across skin is topical delivery, which insures a comfortable, quick and safe method of delivery. The topical drug delivery system is aimed at applying the medication directly on the skin and providing the effectiveness of the formulation directly to the target site [2]. Topical delivery is a more preferable and convenient strategy than other routes of administration owing to its great merits. In addition, it greatly avoids the gastrointestinal problems associated with oral medications as in most cases topical treatment does not provide systemic drug absorption [3]. Numerous drugs could be included in topical formulations in order to cater to a wide variety of purposes such as anti-cancer, anti-oxidant [4], analgesic, anti-inflammatory [5], anti-fungal [6] and anti-bacterial activity [7]. Various pharmaceutical active agents to be delivered topically could be included in different topical therapeutic products such as creams, gels and ointments [8]. Recent technology has been explored for topical delivery that would support the enhancement of their pharmacological effect, termed as nanotechnology [9]. Therefore, in order to maximize the efficacy of the pharmaceutical active agent, it requires its incorporation into the nanocarrier formulation, thus exploiting the concept of nanodrug delivery.

Nanotechnology is a field of science, broadly applied, in which the pharmaceutical active agents are incorporated into nanocarriers [10,11]. These nanocarriers are colloidal systems offering tiny particle size and hence large surface area, which could enhance the solubility and bioavailability of drugs [12]. Additionally, it could avoid drug toxicity and provide great stability for the formulation. Generally, utilization of nanocarriers in the field of drug delivery provides an efficient treatment with minimal adverse reactions. Several nanocarriers are available for effective management of different dermatological disorders; however, lipid-based nanocarriers have displayed great promise in the area of topical delivery [13]. The advantage behind using lipid-based formulations in topical preparations is their aptitude in overcoming the skin barrier [9]. Liposomes, nanoemulsions, nanostructured lipid carriers (NLC) and solid lipid nanoparticles (SLN) are the most commonly used lipid-based nanocarriers [14]. SLN is a prospective nanocarrier system that is applied broadly to provide local effect [15]. A great similarity was observed between nanoemulsions and SLNs, where the oily phase of nanoemulsions was replaced by solid lipid in SLNs [16]. The solid lipid phase supports the nanocarrier with more benefits since it could provide high entrapment of lipophilic drugs. Moreover, SLNs could entrap various vitamins and could be used as a nanolipid carrier for gene delivery. The cost of their manufacturing is low compared to other nanolipid formulations. It could provide extended release for the medication, especially when applied topically [17]. Overall, SLNs possess numerous advantages over other lipid-based nanocarriers, including better stability, enhanced bioavailability of hydrophobic drugs and controlled drug delivery [18]. Therefore, it could be the nanolipid system of choice instead of others. SLNs could be extensively used to entrap various drugs such as anti-cancer [19], anti-inflammatory [20], anti-fungal [21], and anti-bacterial drugs [22].

Anti-bacterial agents are among the mostly applied drug category used for handling several disorders related to bacterial infection [23]. Fusidic acid (FA) is an anti-bacterial drug that comes from a certain fungus known as *Fusidium coccineum* and is recognized for its efficiency in stopping bacterial growth, particularly in the case of ocular and skin infections [7]. It is categorized as a steroid without showing corticosteroid influence [24]. Its anti-bacterial behavior was shown against a wide range of bacterial strains. FA proved to be effective against *Staphylococcus aureus*, which is a common pathogen that could result in various complications [25]. Despite its great efficacy, its poor water solubility represents a great difficulty in its formulation since the solubility in water is about 25.8 μg/mL [26]. Therefore, incorporating FA into nanocarrier systems for improving its solubility and stability is anticipated. The main problem of most anti-bacterial drugs is the development of bacterial resistance, which requires certain combination strategies to overcome such an obstacle [27]. For that reason, the anti-bacterial activity of drugs could be enhanced by combination with other compounds.

The best choice to be incorporated for ensuring great efficiency and safety are the natural products with an anti-bacterial effect. Shea butter is a natural product obtained from the *Vitellaria paradoxa* tree of sub-Saharan Africa, family Sapotaceae [28]. Shea butter has been used widely in food production as well as in cosmetics and for medicinal purposes [29]. The main component of Shea butter is triglycerides that have oleic, linoleic, stearic and palmitic fatty acids in addition to some unsaponifed matter such as tocopherol, sterols and phenols [30]. It was reported that shea itself exhibits extensive influence in treating a wide range of disorders such as fever, diarrhea, rash, skin disorders, wound infections, toothache, stomachache and others [31]. Moreover, shea butter shows different pharmacological activities such as anti-cancer [32], anti-inflammatory [33], anti-oxidant [34], anti-fungal, anti-viral [35] as well as anti-bacterial activity [36,37]. Focusing on its anti-bacterial behavior, shea is active against Gram-positive and Gram-negative bacterial strains, which is attributable to its phytochemical component, including steroids and flavonoids [37].

In regards to providing a product with proper quality attributes, the quality by design (QbD) approach was employed [38]. QbD is a systematic technique that supports the development of a formulation relying on some independent factors that are investigated for their influence on certain responses to reach the best optimized formula. Several methods for optimization could be utilized, such as central composite designs (CCD) technique [39]. Employing CCD would select the optimized formula by studying certain model graphs, statistical analysis and mathematical equations provided by the system [40].

In the forgoing, the goal of the study was to develop an SLN formulation containing FA using shea butter as the lipid phase with anti-bacterial activity. Central composite design was used as a quality by design approach to attain a product with favorable attributes. The optimized preparation was characterized and investigated for its anti-bacterial efficacy.

## 2. Materials and Methods

### 2.1. Materials

Shea butter was provided by NOW^®^ Essential Oils (NOW Foods, Bloomingdale, IL, USA). FA was supplied by Saudi Pharmaceutical Industries & Medical Appliances Corporation (SPIMACO ADDWAEIH, Qassim, KSA). Tween 80 was acquired from ALPHA CHEMIKA (Mumbai, India). MC60 glycerol monocaprylocaprate (Labrafac™) and diethylene glycol monoethyl ether (Transcutol^®^ P) were purchased from Gattefosse SAS (Saint-Priest, Cedex, France). Hydroxypropyl methylcellulose (HPMC) was bought from Sigma-Aldrich Co. (St Louis, MO, USA). All other chemicals were of analytical grade.

### 2.2. Central Composite Factorial Design

CCD was created using some independent factors and their effect on studied responses was investigated. In the current study, CCD used two factors that were studied at two levels (2^2^) to design eleven different run formulations. The independent factors used for the development of SLNs were lipid concentration (A) and surfactant concentration (B). In addition, the studied responses were particle size R_1_ and entrapment efficiency % (EE) R_2_, as displayed in Table 1. Statistical analysis was performed for all data using analysis of variance (ANOVA), in addition to mathematical equations and certain model graphs that help in data interpretation. CCD was generated using Design-Expert version 12.0 software (Stat-Ease, Minneapolis, MN, USA).

### 2.3. Fabrication of FA-SLN

Owing to the great advantages of SLN formulation, such as ease of fabrication and being the most effective carrier for skin delivery, the ultrasonic-nanoemulsification method with certain modifications was applied for the preparation of FA-SLNs as described previously [41]. Concentrations of shea butter and Tween 80 were varied according to the proposed values in CCD. Primarily, a specific amount of shea butter was melted at 50 °C using a water bath and then 100 mg of FA was added. To that lipid phase, 500 mg Labrafac™ and the same amount of Transcutol^®^ P acting as a solubilizer were added and well-mixed until they became homogenous. On the other side, aqueous phase was prepared by heating distilled water containing a definite amount of Tween 80 (up to 10 mL) to the same temperature and then slowly pouring this over the melted lipid phase with continuous stirring to form the pre-emulsion. Homogenization for 5 min at 10.000 rpm using Ultra-Turrax homogenizer (T 25 digital Ultra-Turrax, IKA, Staufen, Germany) followed by 30 s sonication using probe sonicator XL-200, Qsnonica (Newtown, CT, USA) provides FA-SLN with the suitable particle size.

### 2.4. Particle Size and Size Distribution (PDI)

To estimate the particle size and PDI of the developed FA-SLNs, a sample of 5 µL from each formula was diluted to 3 mL distilled water using a disposable cuvette. The measurement was performed utilizing a Malvern zetasizer (Nanoseries, Malvern Instruments, Malvern, UK) through the dynamic light scattering technique that was monitored at 25 °C and angle: 90° [42].

### 2.5. EE%

It is very vital to estimate the percentage of drug entrapped within the formulation; therefore, the centrifugation method described earlier by Elsewedy et al. was followed [43]. A sample of the formulation was added to a centrifugation tube, Amicon^®^ ultra-4 (Ultracel-10K) and centrifuged at 3000 rpm for 1 h at 4 °C. The free drug was collected, diluted and analyzed by spectrophotometer (U.V. Spectrophotometer, JENWAY 6305, Bibby Scientific Ltd., Staffs, UK) at λ_max_ 285 nm. The percentage of EE was calculated from the following equation:

% EE = ((T − F)/T) × 100(1)

where T represents the total drug, while F is the free drug.

### 2.6. Incorporation of FA-SLN in Topical Formulation

Medication intended to be applied topically using viscous formulations provides adequate and easier application over the skin. As a consequence, the optimized FA-SLN formulation was integrated into a preformulated hydrogel preparation. Basically, hydrogel was prepared by gradually sprinkling the gelling agent, 4% HPMC, over 10 mL distilled water and stirring the mixture continuously with a magnetic stirrer (Jeio Tech TM-14SB, Medline Scientific, Oxfordshire, UK) until the clear homogenous hydrogel base was formed. Certainly, gelation of the formulation occurred as a result of decreasing the electrostatic repulsion between the groups of the added gelling agent, which would enhance the helix aggregation and formation of the gel network [44].

### 2.7. Characterization of FA-SLN-Hydrogel

Visual inspection was carried out through examining the final FA-SLN-hydrogel in terms of the final appearance, homogeneity and consistency. In addition, it is very essential for the pH of the formulation to be close to skin pH in order to avoid any skin irritation. Accordingly, a pH meter (MW802, Milwaukee Instruments, Szeged, Hungary) was utilized to determine the pH of the developed FA-SLN-hydrogel. In order to avoid the quick run-off of the topical preparation from the skin, viscosity should be adjusted. FA-SLN-hydrogel was inspected for its viscosity, utilizing the Brookfield viscometer (DV-II+ Pro, Middleboro, MA, USA). The assessment was performed using spindle R5, rotating at 0.5 rpm at room temperature [45]. Moreover, the capability of the topical preparation to spread uniformly and effortlessly when applied over the skin was determined by spreadability measurement. In short, 1 g of the developed hydrogel was placed in between 2 glass slides (25 cm × 25 cm). Next, a 500 g load was placed for 1 min over the slides to apply pressure and allow the formulation to spread. Measuring the spreading diameter represents spreadability [46].

### 2.8. In Vitro Study

ERWEKA dissolution system (ERWEKA, GmbH, Heusenstamm, Germany) was used to determine the percentage of FA released from different formulations, namely: FA suspension, optimized SLN and FA-SLN-hydrogel. A sample of the examined formulation was retained in a glass tube that was covered with cellophane membrane (MWCO 2000–15,000) on one side and attached to the apparatus from the other side. The tubes were held to the apparatus and suspended in media of 500 mL phosphate buffer with pH 5.5 and temperature kept at 32 ± 0.5 °C. The system was operated and adjusted to rotate at 50 rpm. Usually, at definite time intervals, a sample of 3 mL was withdrawn and replaced with an equal volume of fresh buffer. The withdrawn sample was spectrophotometrically assayed at λmax 285 nm with a UV spectrophotometer (JENWAY 6305, Bibby Scientific Ltd., Staffs, UK) [46]. Each experiment was carried out in triplicate with mean value ± SD.

### 2.9. Stability Test

Stability testing was conducted by keeping the prepared FA-SLN-hydrogel in a closed container, stored at two different environments: room temperature (25 ± 2 °C) and refrigeration (4 ± 2 °C). The study continued for 3 months to evaluate the characterization of the formulation, namely: pH, viscosity, spreadability and in vitro release following 1 and 3 months. The study was performed according to the International Conference on Harmonization (ICH) guidelines [45].

### 2.10. In Vivo Study

#### 2.10.1. Animals

Male Wistar rats of average weight 220–250 g were supplied from the Experimental Animal Research Centre at King Saud University, Riyadh, KSA. Rats were kept in the animal house in a controlled environment with dark/light cycle and with free access to food and drink.

#### 2.10.2. Statement of Ethical Approval

All animal experiments were performed in agreement with the guidelines of ethical conduct for animal use at King Faisal University. The protocol of the study was approved by the Research Ethics Committee (REC) of King Faisal University, approval number (KFU-REC/2021-Oct–EA00080).

#### 2.10.3. Skin Irritation

Study of skin irritation provides an indication of the formulation safety. The hair of the rat from the dorsal part was removed using a clipper, one day before going through the study. The examined formulation was spread evenly over the hairless part of the rats. Rats were observed for 7 days after topical application of the formulation for any sign of sensitivity, mainly, irritation, edema or erythema (redness). The responses were interpreted according to a scale range of 0, 1, 2 or 3, which denotes no reaction, minor, moderate and severe erythema with or without edema, respectively [7].

### 2.11. Anti-Bacterial Study

Anti-bacterial activity of FA-SLN-hydrogel was evaluated in comparison to the placebo SLN hydrogel and the marketed FA formulation (Fucidin^®^). The study was conducted using different bacterial strains supplied from American Type Culture Collection (ATCC) such as *Bacillus subtilus* (ATCC 10,400), *Staphylococcus aureus* (ATCC 29,213) and *Klebsiella pneumoniae* (ATCC 10,013). Mueller–Hinton agar was prepared and distributed in a Petri dish for the bacteria to be cultured. Three wells were made in each Petri dish, in which the examined formulation was packed and incubated at 37 ± 1 °C overnight. Afterwards, the inhibition zone diameter was measured as an indicator of the formulation’s anti-bacterial activity. Each experiment was repeated in triplicate with mean value ± SD.

### 2.12. Statistical Analysis

Results were expressed as mean ± standard deviation (SD). Significant difference was detected if *p* < 0.05. Student’s t-test was performed to identify the statistical differences between the groups. All statistical analysis was confirmed by SPSS statistics software, version 9 (IBM Corporation, Armonk, NY, USA).

## 3. Results

### 3.1. Central Composite Factorial Design Validation

Referring to data in Table 1, 11 SLN formulations prepared with shea butter and integrating FA were developed by using particular independent variables and observing their responses. All the data responses were analyzed though ANOVA, which is very important for fitting the design model. It was obvious that the model’s F-value for R_1_ and R_2_ was 622.77 and 729.25, respectively, which implies that the model is significant. In addition, when *p*-values are less than 0.05, it indicates that the model’s terms are significant. In our design, most of the model’s terms were significant. Concerning the lack of fit, it is a very vital value for model fitting. It is known that non-significant lack of fit is much recommended. In our data, lack of fit was 1.07 and 0.5909 with corresponding *p*-values 0.5175 and 0.7387 for R_1_ and R_2_, respectively, which suggests the lack of fit is not significant [47].

### 3.2. Investigating the Dependent Response

#### 3.2.1. Effect of the Independent Factors on Particle Size

As per data in Table 1, particle size of FA-SLN-hydrogel preparations was evaluated and it exhibited values between 216 ± 2.4 and 366 ± 4.6 nm with corresponding PDI value 0.267 ± 0.014 and 0.350 ±0.049. The result demonstrated that the particle size distribution in all formulations was in a narrow range of sizes, which is valuable evidence for formulation stability [48]. Focusing on the data, it was observed that there is a direct relation between particle size and the concentration of the independent factors A and B. Whereas, by increasing concentration of shea butter from 5% to 15%, an increase in the formulation’s particle size was detected as well. The rationale behind that rested on the prospect of the lipid phase to form aggregates and coalescence as it increased the rate of collision that would provide larger particles [49]. Dissimilarly, using higher concentration of Tween 80 would result in particle size diminution upon using the same concentration of lipid phase. The reason behind that could be credited to the role of surfactant in decreasing the interfacial tension at the interface between lipid phase and aqueous phase [50]. Moreover, a layer surrounding the particles might be formed by surfactant, which functions like a barrier that protects the particles from being aggregated and forming a larger size [51]. The previous findings were confirmed by a mathematical equation created by the design software since there was a positive sign in front of factor A indicating parallel effect while the negative sign in front of factor B pointed toward its antagonistic influence on the studied response R_1_:

R_1_ = 273.789 + 58 A − 18.5 B − 4.25 AB + 9.52632 A^2^ + 4.02632 B^2^ (2)

**Figure 1:**
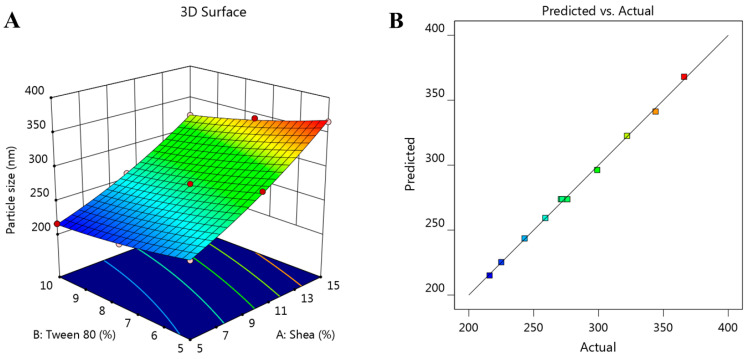
(**A**) The 3D-response surface graph showing the influence of independent factors, shea and Tween 80 concentrations, on the investigated particle size (R_1_), and (**B**) linear correlation plot between predicted and actual values validating the effect of various independent factors on particle size response (R_1_).

Further, several graphs were generated from the software, confirming the attained influence of the selected independent factors on response R1. Figure 1A shows a 3D-response surface graph that confirms the positive relation between factor A and the particle size of the fabricated FA-SLN formulations while confirming the negative one between factor B and the response. Additionally, Figure 1B illustrates the linear correlation between the predicted and the actual values of the response since the values of the predicted R^2^ (0.9899) and adjusted R^2^ (0.9968) are very close to each other, which points to a reasonable agreement between each other.

#### 3.2.2. Effect of the Independent Factors on EE

Entrapment of FA within SLNs was estimated via determining the % of EE that appeared to be ranged between 57.0 ± 2.0 and 88.0 ± 2.2%. It was remarkable that the selected independent factors prominently affect the % of EE (R_2_). It is highly obvious from Table 1 that increasing lipid phase concentration would positively affect the EE, thus using higher concentrations of shea would result in a corresponding increase in R_2_. This outcome was presumed to be due to the lipophilicity of FA, hence, an improvement of its solubility in the melted lipid phase was perceived, which in turn enhanced the drug entrapment [52]. Additionally, increasing lipid phase concentration resulted in higher particle size that could accommodate larger amounts of the drug due to space availability [53]. Contrariwise, an indirect relation was detected between independent factor B and the EE response, hence increasing surfactant concentration would decrease the amount of drug entrapped. This definitely could be attributed to the mentioned fact that higher concentration of surfactant would form a small particle size, which could not accommodate higher entrapment. The obtained mathematical equation explains and confirms the observed result since factor (A) supported by a positive sign revealed a positive synergistic effect. However, factor B carried a negative sign that represents the opposite influence:

R_2_ = 72.6364 + 12.5 A − 3.5 B (3)

**Figure 2:**
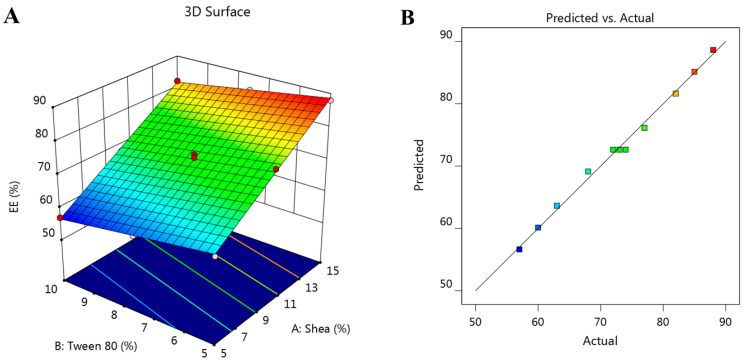
(**A**) The 3D-response surface graph showing the influence of independent factors, shea and Tween 80 concentrations, on the investigated EE (R_2_), and (**B**) linear correlation plot between predicted and actual values validating the effect of various independent factors on EE response (R_2_).

Added to that, the generated 3D-response surface plot that is shown in Figure 2A further clarifies the noticeable influence of the independent factors A and B on the studied response R_2_. Moreover, Figure 2B emphasizes the linear correlation between predicted versus actual values related to EE. In the same manner, the linear correlation between predicted and actual values was affirmed because there was a reasonable agreement between predicted and adjusted R^2^ values, which were 0.9903 and 0.9932, respectively. It was noted that the difference between both values was less than 0.2, which confirms the observed relation.

### 3.3. Confirming the Optimization

**Figure 3:**
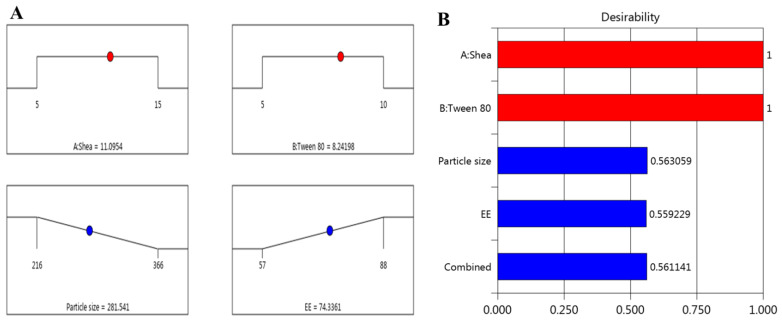
(**A**) Optimization ramps for the independent factors along with the predicted values of responses; (**B**) plot showing the desirability value of all independent factors.

**Figure 4:**
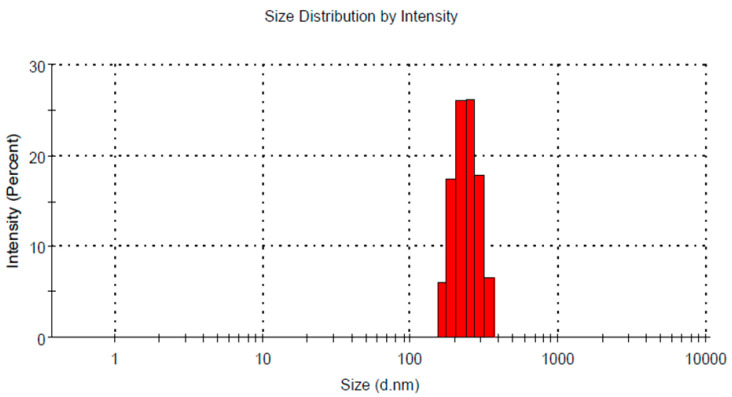
Particle size of optimized FA-SLN formulation.

Based on the gathered data, and following the numerical optimization standards, the optimized formulation of FA-SLN was selected by directing the responses toward certain relevant criteria in order to achieve the higher formulation attributes. The independent factors were adjusted to be in range; however, the responses were fitted to minimizing the particle size and maximizing the EE. Figure 3A shows the ramps obtained from the report of the numerical optimization in the design. It displays the suggested concentrations of the independent variables expected to provide the optimized formula that is composed of 11.09% shea butter and 8.23% Tween 80. Several solutions were presumed; the one with the highest desirability value was selected (0.561), as seen in Figure 3B. Based on that assumption, a new FA-SLN formulation was fabricated, representing the optimized formula where a close similarity was observed between the predicted and the observed values as apparent in Table 2. The particle size was found to be 283.83 nm and the EE was 73.057%. Figure 4 presents the particle size of the optimized FA-SLN.

### 3.4. Characterization of FA-SLN-Hydrogel

According to the concerns of the optimization process, the optimized SLN containing FA was fabricated and then was included in the preformulated HPMC hydrogel base. The resultant product would be the FA-SLN-hydrogel that seems to be stable with no hint of phase separation, which would be evaluated in terms of a topical preparation. Visual examination of the developed FA-SLN-hydrogel was executed and it was found to be a whitish, smooth and homogenous preparation. Regarding pH measurement, it is an essential parameter in characterizing the topical formulation in order to confirm whether it could be an irritant or not. Consequently, pH of the FA-SLN-hydrogel was evaluated to be 6.2 ± 0.21, which seemed to be close to the pH of the skin, suggesting the safety of the formula and suitability for topical application [54]. As well, viscosity of the formulation was considered a good and reliant parameter that could determine the extent of drug diffusion from the formulation and could affect the drug’s in vitro behavior [55]. The viscosity of the FA-SLN-hydrogel was estimated to be 15,610 ± 927 cP, which is expected to be distinctive and in the acceptable range for topical application [56]. As mentioned, the importance of spreadability lies in detecting how easily the formulation would spread over the affected area upon topical application, which achieves convenience for patients. Spreadability of the FA-SLN-hydrogel was calculated as 51.1 ± 2.4 mm, which is considered typical for topical formulations [57].

### 3.5. In Vitro Release

**Figure 5:**
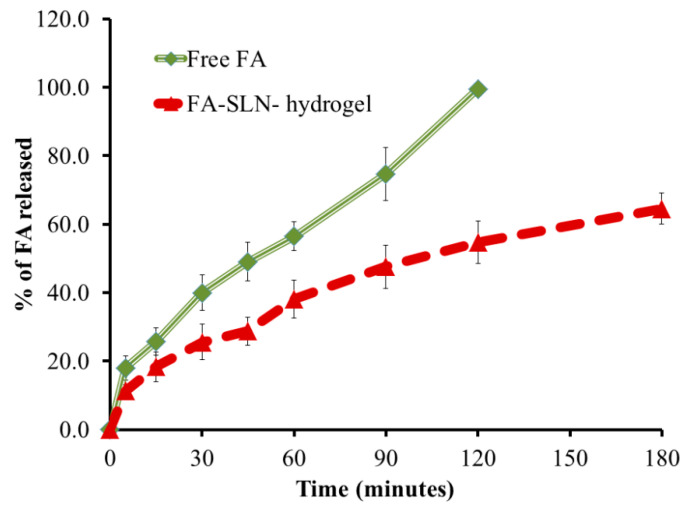
In vitro release study of FA from FA-SLN-hydrogel formulation using phosphate buffer saline pH 5.5 at 32 ± 0.5 °C.

In vitro release of FA from the fabricated SLN-hydrogel compared to free FA was executed using phosphate buffer pH 5.5 and adjusted at 32 ± 0.5 °C to mimic the skin conditions; the profile of the investigation is outlined in Figure 5. It is obvious that the dissolution of FA could reach 56.43 ± 4.18 after 60 min and there could be complete dissolution following 120 min (99.5%). However, the release from SLN was 38.5 ± 5.5, 54.7 ± 6.3 and 64.6 ± 4.5% after 60, 120 and 180 min, respectively, when released from the SLN-hydrogel formulation. Certainly, FA was incorporated into the SLN formula and remained in the internal lipid phase, and then was included in the hydrogel base that caused the formulation to take a longer time to be released and pass through many layers [58]. Secondly, the viscosity of the preparation would play a vital role in slowing down the rate of drug release [59].

### 3.6. Stability Study

**Figure 6:**
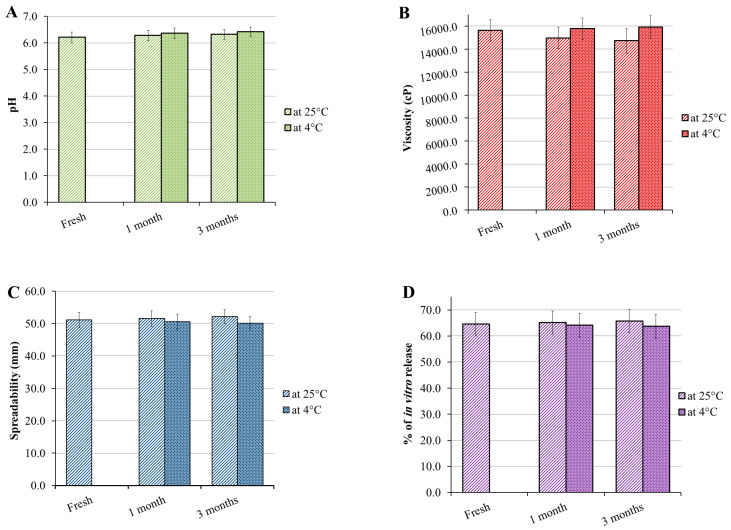
Graphs representing stability testing of FA-SLN-hydrogel following 1 and 3 months storage at 25 ± 2 °C and 4 ± 2 °C in terms of (**A**) pH, (**B**) viscosity, (**C**) spreadability and (**D**) in vitro study when compared with fresh formulation.

Certain parameters, mainly, pH, viscosity, spreadability and in vitro release were evaluated to investigate the physical stability of the FA-SLN-hydrogel formulation. The study was accomplished using stored formula at two different conditions: 25 ± 2 °C and 4 ± 2 °C for a period of 1 and 3 months and the results are displayed in Figure 6. The overall results denoted that there were no significant variations in all assessed parameters at both conditions for the whole period of storage when compared to fresh formula. The obtained results highlighted the stated fact previously investigated for SLN and proved its stability owing to the lipid core in its structure that was covered with surfactant, which contributes to greater stability upon storage [60].

### 3.7. Skin Irritation

Irritation reactions that might be observed on rats treated with FA-SLN-hydrogel were detected by implementing a skin irritation study. The dorsal area at which the formula was smeared was checked for 7 days and it was remarkable that the results scored 0, which means that no irritation reactions such as edema or erythema were apparent throughout the whole period of the study.

### 3.8. Anti-Bacterial Study

**Figure 7:**
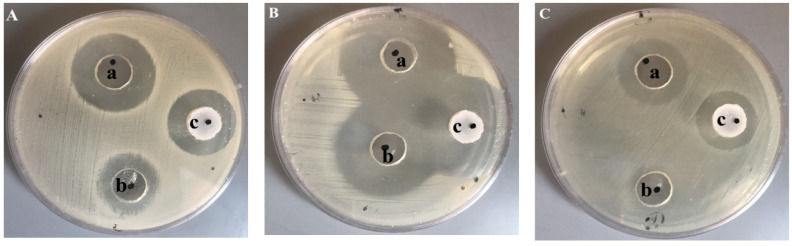
Inhibition zone caused by examined formulations: (a) FA-SLN-hydrogel, (b) placebo SLN-gel and (c) marketed FA (Fucidin^®^) on different bacterial strains: (**A**) *Bacillus subtilis*, (**B**) *Staphylococcus aureus* and (**C**) *Klebsiella pneumonia*.

Anti-bacterial study was implemented against different bacterial strains in order to assess the anti-bacterial effect of the FA-SLN-hydrogel formulation. The study depended on measuring the diameter of the inhibition zone that could appear as a result of formulation activity counter to the bacteria. The inhibition zone of different formulations, namely FA-SLN-hydrogel, placebo SLN-hydrogel and marketed product (Fucidin^®^) can be observed from Table 3 and Figure 7. It was observable that FA-SLN-hydrogel exhibited a distinctive anti-bacterial activity against *Bacillus subtilis, Staphylococcus aureus,* and *Klebsiella pneumoniae* since it demonstrated a significant inhibition zone when compared to placebo SLN-hydrogel and Fucidin^®^ (*p* < 0.05). It was noteworthy that placebo SLN-hydrogel showed remarkable bacterial growth inhibition, which indicates that the anti-bacterial activity could be credited to shea butter itself. That finding emphasized the valuable role of shea butter as an anti-bacterial agent [31,36]. This is in addition to confirming its broad spectrum activity against a wide range of Gram-positive and Gram-negative bacteria as stated previously [36,37]. With regard to our findings, the higher anti-bacterial activity demonstrated by the FA-SLN-hydrogel formula could be ascribed to combining FA and shea butter, which resulted in enhancement of the anti-bacterial activity of FA. That means that the anti-bacterial activity of shea butter could improve the activity of FA in preventing bacterial growth.

## 4. Conclusions

The present study investigated the development of SLN formulation using shea butter as a lipid phase incorporating fusidic acid. Central composite factorial design was applied as a quality by design approach for developing 11 formulations depending on shea and Tween 80 concentrations to observe their effect on particle size, shown to be 283.83 nm and 73.057%. For better topical application, the optimized FA-SLN was integrated into HPMC-hydrogel base. FA-SLN-hydrogel was examined for its physical character and revealed satisfactory pH (6.2), viscosity (15610 cP) and spreadability (51.1 mm) that seemed to be adequate for topical application. The formulation was stable and safe without any sign of irritation. Furthermore, shea butter displayed prominent anti-bacterial activity that could potentiate the effect of FA in inhibiting bacterial growth. Finally, SLN could be suggested as a distinctive nanolipid formulation anticipated for topical application.

## Figures and Tables

**Table 1:** Formulations of FA-SLN offered by CCD with their detected responses.

Formula	Independent Factors		Dependent Responses		PDI
	A (%)	B (%)	R_1_ (nm)	R_2_ (%)	
F1	5	5	243 ± 2.6	63 ± 1.1	0.340 ± 0.035
F2	15	7.5	344 ± 3.6	85 ± 2.0	0.348 ± 0.034
F3	10	7.5	271 ± 2.0	72 ± 2.6	0.350 ± 0.049
F4	15	5	366 ± 4.6	88 ± 2.2	0.340 ± 0.031
F5	15	10	322 ± 4.0	82 ± 2.3	0.276 ± 0.018
F6	10	7.5	276 ± 3.1	74 ± 3.0	0.338 ± 0.027
F7	5	7.5	225 ± 2.3	60 ± 2.4	0.333 ± 0.028
F8	10	7.5	272 ± 2.5	73 ± 2.5	0.267 ± 0.014
F9	5	10	216 ± 2.4	57 ± 2.0	0.319 ± 0.019
F10	10	10	259 ± 3.6	68 ± 2.2	0.323 ± 0.035
F11	10	5	299 ± 3.5	77 ± 2.1	0.275 ± 0.010

A: shea butter concentration; B: Tween 80 concentration; R_1_: particle size; R_2_: EE.

**Table 2:** Predicted versus observed value of the optimized FA-SLN formulation.

Independent Selected Factor	Constraint	
Shea butter concentration (%)	In range	
Tween 80 concentration (%)	In range	
**Response**	**Predicted values**	**Observed values**
Particle size (nm)	281.541 ± 2.69	283.83 ± 3.75
EE (%)	74.336 ± 0.83	73.057 ± 2.51

**Table 3:** Microbiological activity of examined formulations counter to different bacterial strains.

Bacterial Type	Inhibition Zone (cm)		
	FA-SLN-Gel	Placebo-SLN-Hydrogel	Marketed FA (Fucidin^®^)
*Bacillus subtilis*	3.04 ± 0.14 *#	2.05 ± 0.14 *	2.73 ± 0.11 #
*Staphylococcus aureus*	4.27 ± 0.11 *#	3.84 ± 0.11 *	4.02 ± 0.10 #
*Klebsiella pneumoniae*	2.52 ± 0.13 *#	2.14 ± 0.15 *	1.12 ± 0.11 #

Values are expressed as mean ± SD, *n* = 3. * (*p* < 0.05) compared to Fucidin^®^; and # (*p* < 0.05) compared to placebo formulation.

## Data Availability

Not applicable.
